# The Effect of Cluster Size Variability on Statistical Power in Cluster-Randomized Trials

**DOI:** 10.1371/journal.pone.0119074

**Published:** 2015-04-01

**Authors:** Stephen A. Lauer, Ken P. Kleinman, Nicholas G. Reich

**Affiliations:** 1 Division of Biostatistics and Epidemiology, School of Public Health and Health Sciences, University of Massachusetts, Amherst, MA, USA; 2 Department of Population Medicine, Harvard Medical School/Harvard Pilgrim Health Care Institute, Boston, MA, USA; University of Westminster, UNITED KINGDOM

## Abstract

The frequency of cluster-randomized trials (CRTs) in peer-reviewed literature has increased exponentially over the past two decades. CRTs are a valuable tool for studying interventions that cannot be effectively implemented or randomized at the individual level. However, some aspects of the design and analysis of data from CRTs are more complex than those for individually randomized controlled trials. One of the key components to designing a successful CRT is calculating the proper sample size (i.e. number of clusters) needed to attain an acceptable level of statistical power. In order to do this, a researcher must make assumptions about the value of several variables, including a fixed mean cluster size. In practice, cluster size can often vary dramatically. Few studies account for the effect of cluster size variation when assessing the statistical power for a given trial. We conducted a simulation study to investigate how the statistical power of CRTs changes with variable cluster sizes. In general, we observed that increases in cluster size variability lead to a decrease in power.

## Introduction

The cluster-randomized trial (CRT) is a common study design in public health research, in which interventions are administered to groups rather than to individuals. In situations where dividing a group of individuals into treatment and controls is unethical or impossible, a CRT design retains many of the strengths of an individually randomized study design [1]. By comparing the outcomes of small populations (clusters), CRTs can observe the impacts of interventions on a community as a whole.

**Fig 1:**
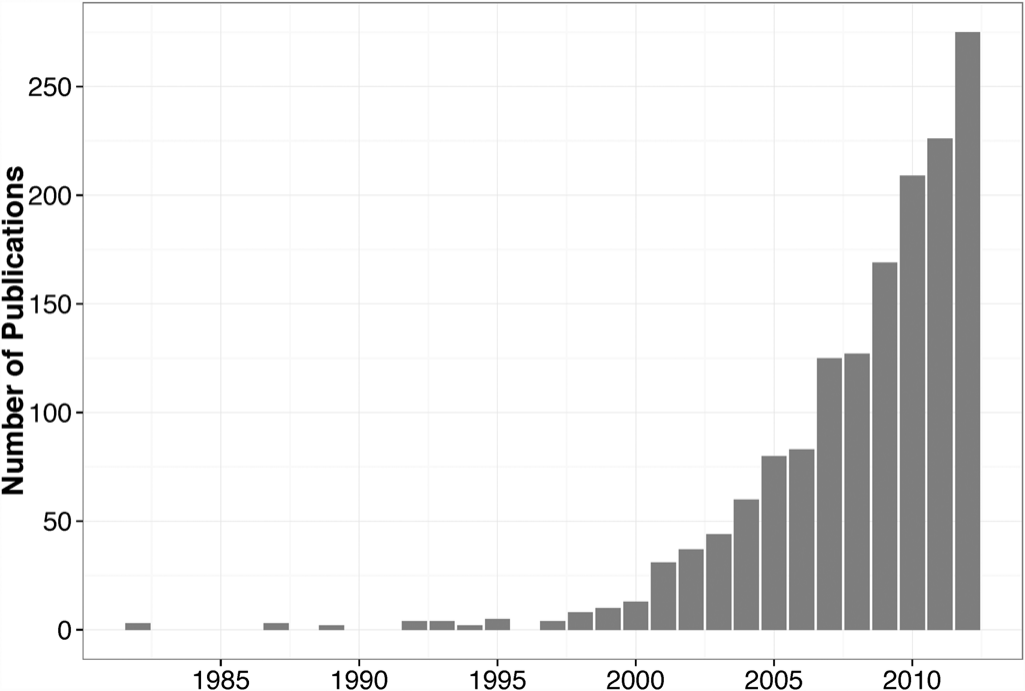
The annual number of articles published on CRTs (either trials themselves or methods for trials) has increased from 4 to 275 since 1997. Source: Web of Science. Search syntax used: (TI = (“cluster random*”) OR TI = (“group random*”)) AND Document Types = (Article).

The number of published articles utilizing CRTs has increased every year since 1997 (See Fig. 1). Due to its rising popularity, this relatively complex study design is facing greater scrutiny from the scientific community. The Consolidated Standards of Reporting Trials (CONSORT) Group issued guidelines for conducting CRTs in 2004 [2], with an update published in 2012 [3]. One important component of CRT design is the sample size calculation; in which researchers must find the correct number of clusters to achieve sufficient statistical power. In *CONSORT 2010 statement: extension to cluster randomised trials*, there was an added focus on sample size reporting, which included a note about accounting for varying cluster sizes.

This has been a subject of interest since Donner and Klar’s seminal paper *Randomization by cluster: sample size requirements and analysis* in 1981 [4]. As detailed in *Unequal cluster sizes for trials in English and Welsh general practice: implications for sample size calculations* [5], Kerry and Bland derived a formula to calculate the design effect of a CRT based on the number of participants in each cluster. The design effect is the ratio of the sample size required for a CRT over that of an individually randomized trial with the same power. Eldridge et al built upon this formula so that design effect could be calculated knowing only the mean cluster size and the coefficient of cluster size variation [6, 7].

Another approach to accounting for cluster size variation is by using a measure of relative efficiency [8–11]. Relative efficiency is mathematically derived and easy to implement once computed. However, there are several ways to calculate relative efficiency, all of which use complicated methods that may create obstacles to their use in practice. We seek to simplify the process of estimating the needed sample size for a CRT in the presence of variable cluster sizes. We hope this effort will encourage continued improvement in efficient implementation of CRTs within the medical and social science communities.

Using the statistical programming language R v3.02 [12] and the package clusterPower [13, 14], we designed a controlled simulation experiment in which we simulated hundreds of thousands of hypothetical CRTs. Using the results from these simulations, we examined the effect of variability in cluster sizes on statistical power of CRTs and developed simple and concrete quantitative guidelines for researchers who design CRTs with high variability in cluster sizes. This manuscript file was typeset reproducibly using the R package knitr [15, 16], which we used to call files of pre-processed results from the simulation studies. All code and data for this project is available at https://bitbucket.org/nickreich/clustersizepaper.

## Project Overview

We developed a framework that allowed us to measure the impact that variability in cluster size has on the power of a CRT. This framework was built on the foundation of the clusterPower package in R [13]. Statistical power is defined as the probability of rejecting the null hypothesis given that the null hypothesis is not true. In brief, to estimate statistical power for a given CRT design, we stochastically simulated data (i.e. results from a trial) from a hypothetical CRT design with a known, non-zero intervention effect size. Among all simulated trials, the percentage of time the null hypothesis is rejected is therefore an accurate estimate of the power for this design. We have leveraged this simple simulation framework to answer a complex question about CRT study design: how does variability in cluster size impact the power of a CRT?

We designed a simulation study to systematically gather data on how cluster size variability impacts power and sample size requirements for a CRT. To provide a focused study of the effect of cluster size variability on CRT sample size, we limited the current investigation to a common CRT design. Namely, we focused on equal-armed CRTs that had a continuous outcome measure (as opposed to binary or count data) and assumed that the design did not incorporate a “controlled comparison” (e.g. a crossover, baseline comparison, or matching). We used a data generating model similar to that given by Reich et al. [13]:

$$\begin{array}{ccc} Y_{jk}|X_{k} & = & \eta_{k}+\Delta \cdot X_{k}+\epsilon_{jk},\\ \eta_{k} & ∼ & \mathrm{Normal}(0,\sigma_{\eta }^{2}),\\ \epsilon_{jk} & ∼ & \mathrm{Normal}(0,\sigma_{\epsilon }^{2}). \end{array}$$ (1)

where *Y*_*jk*_ is the observed outcome for person *j* (*j* = 1, …, *J*_*k*_) in cluster *k* (*k* = 1, …, *K*; *K* = total number of clusters), *X*_*k*_ is a binary variable indicating whether cluster *k* was assigned to the treatment (*X*_*k*_ = 1) or control (*X*_*k*_ = 0) group, *Δ* is the non-standardized treatment effect size, *η*_*k*_ is the effect due to variation between clusters, and *ε*_*jk*_ is the effect due to variation between the individuals in each cluster.

### Simulation study

The following list provides a brief description of each step in our simulation study. We provide a more detailed explanation of each step in the next section.

1. Defined the *parameter sets* (***θ***_***i***_). Each ***θ***_***i***_ is a vector of variables used to calculate the statistical power (*P*) of a theoretical CRT. The following components make up ***θ***_***i***_ and are also listed (with the values we assumed for each) in Table 1:
  - Type I Error (*α*, fixed at 0.05 for all experiments)
  - Mean cluster size (*μ*)
  - Intraclass correlation coefficient ($\text{ICC}=\frac{\sigma_{\eta }^{2}}{\sigma_{\eta }^{2}+\sigma_{\varepsilon }^{2}}$)
  - Between cluster variance ($\text{BCV}=\sigma_{\eta }^{2}$)
  - Number of clusters needed to reach 80% power with fixed cluster sizes (*C* ^80^)
  - Effect size (*Δ*, calibrated on the combination of above variables)
  Using parameters (i-v) and a type II error (*β*) of 0.2 in Murray’s effect size equation (defined in Methods), we calculated the minimum effect size required to find a significant result in a properly powered CRT, *Δ*. By jointly varying parameters (i-v) over realistic ranges of values, we created carefully calibrated hypothetical CRT settings. Then, when we simulated data from CRTs in these setting to make power estimates with clusterPower, we allowed for the actual number of clusters in the study (*C* ^*A*^) to be different than *C* ^80^.
2. Estimated the statistical power when cluster sizes were fixed at *μ*. We refer to these estimates as our *fixed cluster size power estimates*, ${\hat{P}}_{\theta_{i}}^{F}(C^{A})$. To do this, we simulated hypothesis tests on 5000 unique datasets for each (***θ***_***i***_, *C* ^*A*^) pair.
3. Estimated the statistical power when cluster sizes have variability, defined by several coefficient of variation (*cv*) levels. We refer to these estimates as *variable cluster size power estimates*, ${\hat{P}}_{\theta_{i}}^{cv}\left(C^{A}\right)$. To do this, we generated *S* = 2000 *variable cluster size sets* for each (***θ***_***i***_, *C* ^*A*^, *cv*) combination and ran a simulation on each variable cluster size set, which output a dataset. Hypothesis testing was performed on each dataset and produced a ${\hat{P}}_{\theta_{i},s}^{cv}\left(C^{A}\right)$, where *s* = 1, …, *S*. The average result over all *S* datasets defined the ${\hat{P}}_{\theta_{i}}^{cv}(C^{A})$.
4. Calculated the required number of clusters for each combination of ***θ***_***i***_ and *cv*. The vectors ${\hat{\text{P}}}_{\theta_{i}}^{\text{F}}$ and ${\hat{\text{P}}}_{\theta_{i}}^{\text{cv}}$ form power curves for each ***θ***_***i***_ along the range of *C* ^*A*^. Using the values just above and below *P* = 0.8, we interpolated the point where *C* ^*A*^ is equal to the number of clusters required to achieve a statistical power of 80% for fixed-size cluster sets (${\hat{C}}_{\theta_{i}}^{F}$, which approximates *C* ^80^) and variable cluster sets (${\hat{C}}_{\theta_{i}}^{cv}$).
5. Using our results, we observed the effect of *cv* on the required number of clusters (${\hat{C}}_{\theta_{i}}^{cv}$).

**Table 1:** List of simulation parameters and their values.

	**Simulation Parameter**	**Values**								
***θ***_***i***_	Type I Error (*α*)	.05								
	Mean Cluster Size (*μ*)	20	50	75	100	125				
	ICC	.001	.002	.006	.01	.03	.08	.2		
	BCV	.01	.1	1						
	Number of clusters required to reach 80% power (*C* ^80^)	10	20	40	60					
	Effect Size (*Δ*)	Calibrated on the combination of the above variables								
	Actual No. of Clusters (*C* ^*A*^)	5	10	15	20	40	60	80*	100**	120**
	Fixed-Variance Sims	5000								
	Coefficient of Variation (*cv*)	0.5	1.0	1.5						
	Variable-Sized Sets (*S*)	2000								
	Variable-Sized Set Sims	1								

* Only used when *C* ^80^ > 10

** Only used when *C* ^80^ > 20

## Methods

### Step 1: Parameter Selection

To test the impact of cluster size variability on *P*, we simulated from 420 parameter sets (***θ***_***i***_). This is the number of unique combinations of *μ* values (5), ICC values (7), BCV values (3), and *C* ^80^ values (4). Each ***θ***_***i***_ was simulated across a range of *C* ^*A*^ values (6 to 9, depending on *C* ^80^) to create a total of 3,255 data points.

For each ***θ***_***i***_ parameter set, we calculated a *Δ* value based on *α*, *β*, *μ*, ICC, BCV, and *C* ^80^ according to these equations [17]:

$$\begin{array}{ccc} \Delta & = & \sqrt{\frac{4\cdot \left(\sigma_{\epsilon }^{2}+\mu \cdot \text{BCV}\right){\left(t_{\alpha /2}+t_{\beta }\right)}^{2}}{\mu \cdot C^{80}}},\\ \text{where:}\;\sigma_{\epsilon }^{2} & = & \frac{\text{BCV}\cdot (1-\text{ICC})}{\text{ICC}}. \end{array}$$ (2)

Parameter selection required balancing the desire for well-spaced values across a meaningful range for each parameter with the computational burden of the simulations. (Over 68 million simulations took approximately two weeks running in parallel in 21 threads on a 12-core Mac Pro Desktop.)

The values for *α* and *β* were set at the standard type I and type II error rates of 0.05 and 0.2, respectively.

The five values of the mean cluster size, *μ*, were distributed between 20 and 125. We limited the maximum *μ* to 125 because simulations with large cluster sizes take considerably more computational time.

The ICC quantities were seven evenly-spaced, rounded log-linear values between 0.001 and 0.2.

The three BCV values were equally spread out on a log scale, spanning from 0.01 to 1.

The four *C* ^80^ values were chosen based on the range of popular numbers of clusters for CRTs. A CRT with 10 clusters would constitute a small trial and a CRT with 60 clusters is decidedly larger. We set the maximum *C* ^80^ value at 60 because larger quantities of clusters take significantly longer to simulate.

The *C* ^*A*^ values ensured that the power curves for each CRT ranged from near 0 to 1. Initial tests showed that a wide range (5 ≤ *C* ^*A*^ ≤ 120) was needed to accomplish this when *C* ^80^ = 60. However, for smaller *C* ^80^ values, the statistical power reached 1 sooner and thus the larger values of *C* ^*A*^ were unnecessary.

### Step 2: Generate fixed cluster size power estimates

Prior to investigating the effect of variable cluster sizes on statistical power, we calibrated the empirical estimates of power from the clusterPower package against the formula-based estimates of power (2). Since existing CRT formulas assume equal cluster sizes, we ran one 5000-simulation fixed cluster size CRT for each (***θ***_***i***_, *C* ^*A*^) pair. This produced *fixed cluster size power estimates*, ${\hat{P}}_{\theta_{i}}^{F}(C^{A})$, where every cluster size in each CRT was fixed at *μ*. In Step 4, these estimates are used to compare the difference between the estimated number of clusters needed based on formulaic and simulated power calculations.

### Step 3: Generate variable cluster size power estimates

Next, we generated *variable cluster size sets*, sets of *C* ^*A*^ randomly-drawn cluster sizes from a negative binomial distribution with mean *μ*. The negative binomial distribution was chosen due to the fact that the cluster sizes from trials that study investigators have worked on have shown over-dispersion similar to that of these distributions. We fixed the coefficient of variation (*cv*) of the negative binomial distribution, to roughly match observed skewness in cluster sizes from published CRTs. We used three levels of variability in cluster sizes: low variance (*cv* = 0.5), mid variance (*cv* = 1.0), and high variance (*cv* = 1.5).

In our high variance cluster size set, the *cv* was fixed at 1.5, which is about twice the size of the largest *cv* in other CRT papers [8–10]. This ensured that our high variance cluster size power estimates represented a near-upper bound on the number of variable-sized clusters required in practice. By holding the *cv* constant, the size parameter of the negative binomial distribution (*r*) and consequently the variance, are functions of *μ* [18]:

$$\begin{array}{ccc} r & = & \frac{\mu }{\mu \cdot cv^{2}-1}. \end{array}$$ (3)

**Fig 2:**
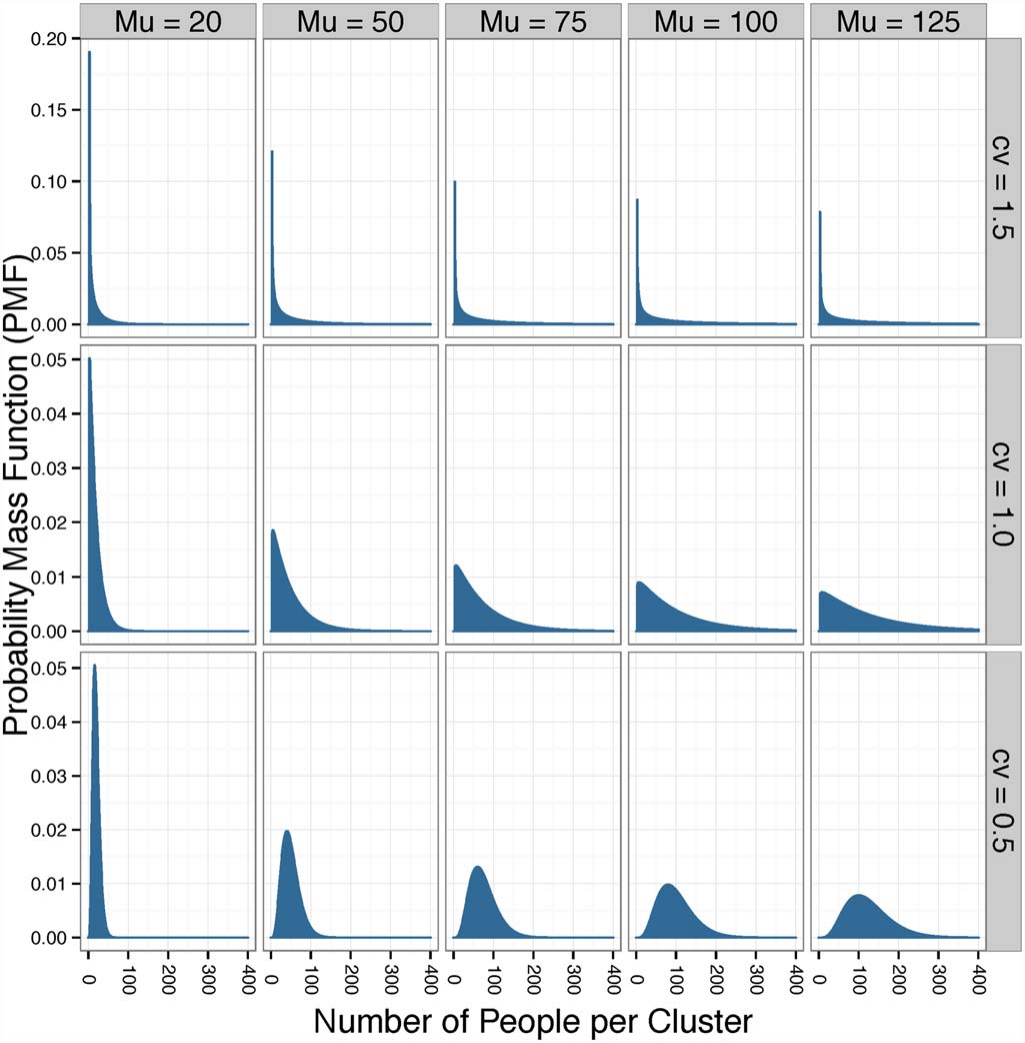
The negative binomial distributions used to draw the cluster sizes sets for each mean cluster size (*μ*) and coefficient of variance (*cv*). Each set had between 5 and 120 clusters, which made up the sample size of a CRT.

When *cv* = 1.5, draws from a negative binomial distribution with a mean of 20 yield a value of zero 18% of the time, a value of fifty or greater 12% of the time, and a value of 140 or greater 1% of the time. Because a cluster cannot consist of zero participants, we set the minimum cluster size to 3. Specifically, to obtain a mean cluster size of 20, we drew from a negative binomial distribution with a mean of 17 before adding 3 to all cluster sizes. The probability mass functions that these distributions are drawn from are shown in Fig. 2.

The number of participants in the variable cluster size sets fluctuated immensely. Since this has an effect on the estimated power of a CRT, many variable cluster size sets (*S* = 2000) were created for each (***θ***_***i***_, *C* ^*A*^) pair, so that the mean cluster size of all sets would converge to *μ*. We ran one clusterPower hypothesis test simulation for every variable cluster size set. This generated a collection of binary outcomes (1 if the null hypothesis was correctly rejected, 0 if not), ${\hat{P}}_{\theta_{i},s}^{cv}(C^{A})$ for *s* = 1, ⋯, *S*. These outcomes were averaged to create one point:

$$\begin{array}{ccc} {\hat{P}}_{\theta_{i}}^{cv}\left(C^{A}\right) & = & \frac{\sum_{s=1}^{S}{\hat{P}}_{\theta_{i},s}^{cv}\left(C^{A}\right)}{S}. \end{array}$$ (4)

### Step 4: Estimate number of clusters needed with and without variability

Let $\hat{P}$ denote any ${\hat{\text{P}}}_{\theta_{i}}^{\text{F}}$ or ${\hat{\text{P}}}_{\theta_{i}}^{\text{cv}}$, a vector of power values for each of the ***θ***_***i***_ across *C* ^*A*^, with or without variance. This vector creates a power curve that allows us to estimate the number of clusters required for that parameter set at a certain level of variability.

For each $\hat{P}$, we found the $\hat{P}(C^{A})$ and *C* ^*A*^ at the points just below and above *P* = 0.8. ${\hat{P}}_{0.8^{-}}$ and *C*_0.8^−^_ are the power and number of clusters below our point of interest. ${\hat{P}}_{0.8^{+}}$ and *C*_0.8^+^_ are the power and number of clusters above our point of interest. ${\hat{P}}_{0.8^{-}}$ and ${\hat{P}}_{0.8^{+}}$ were placed in a vector and *C*_0.8^−^_ and *C*_0.8^+^_ into a matrix as follows:

$$\begin{array}{ccc} \left[\begin{array}{c} {\hat{P}}_{0.8^{-}}\\ {\hat{P}}_{0.8^{+}} \end{array}\right] & = & \left[\begin{array}{cc} C_{0.8^{-}} & 1\\ C_{0.8^{+}} & 1 \end{array}\right]\cdot \left[\begin{array}{c} \hat{a}\\ \hat{b} \end{array}\right]. \end{array}$$ (5)

In solving this equation, we find the slope ($\hat{a}$) and intercept ($\hat{b}$) of a line that passed between the points (*C*_0.8^−^_, ${\hat{P}}_{0.8^{-}}$) and (*C*_0.8^+^_, ${\hat{P}}_{0.8^{+}}$). From there, we set *P* = 0.8 to find the number of clusters required to achieve sufficient power, $\hat{C}$:

$$\begin{array}{ccc} 0.8 & = & \hat{a}\cdot \hat{C}+\hat{b}. \end{array}$$ (6)

Where *Ĉ* is the estimated number of required clusters to achieve a statistical power of 0.8. When using ${\hat{\text{P}}}_{\theta_{i}}^{\text{F}}$, $\hat{C}={\hat{C}}_{\theta_{i}}^{F}$, which is a simulated estimate of *C* ^80^. For ${\hat{\text{P}}}_{\theta_{i}}^{\text{cv}}$, $\hat{C}={\hat{C}}_{\theta_{i}}^{cv}$, which is a value for which Murray’s formulas (2) cannot be used. For the sake of simpler notation, we will refer to these as ${\hat{C}}^{F}$ and ${\hat{C}}^{\mathrm{cv}}$.

**Fig 3:**
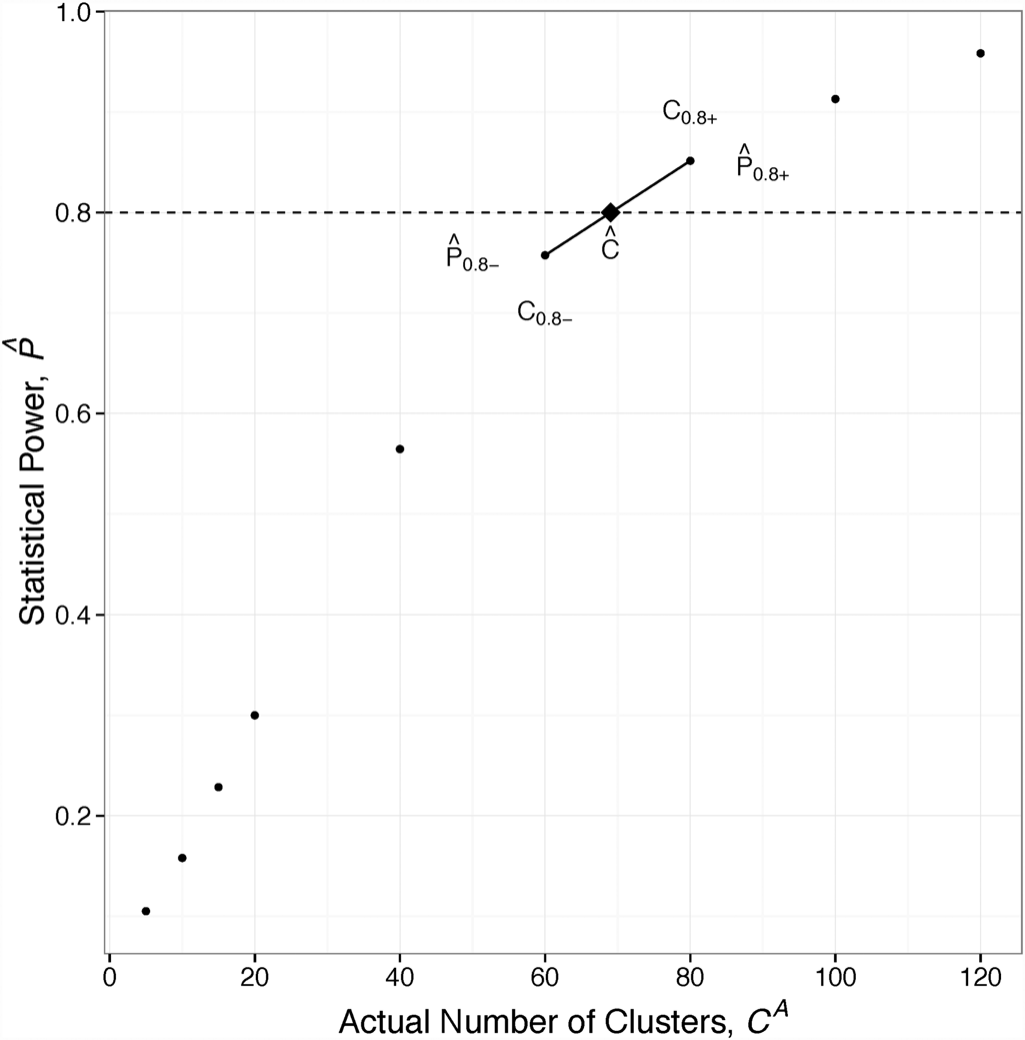
How to find the required number of clusters (*Ĉ*). First, for a given set of parameters, ***θ***_***i***_, hypothesis test simulations are run for each level of *C* ^*A*^. Second, the results of the simulations are averaged into one point per level. Third, $\hat{C}$ is calculated by interpolating a point at $\hat{P}=0.8$ between (*C*_0.8^−^_, ${\hat{P}}_{0.8^{-}}$) and (*C*_0.8^+^_, ${\hat{P}}_{0.8^{+}}$.

A graphical representation of this process is shown in Fig. 3.

Additionally, we defined and compared the percentage change in the number of clusters needed to achieve 80% power between those required by the Murray equation and the variable cluster size sets as:

$$\begin{array}{ccc} {\hat{C}}^{cv}\% & = & \frac{{\hat{C}}^{cv}-C^{80}}{C^{80}}. \end{array}$$ (7)

### Step 5: Analyze effects of cluster size variance

With ${\hat{C}}^{\mathrm{cv}}$, ${\hat{C}}^{F}$, and *C* ^80^, we observed the effect of cluster size variance on the required number of clusters.

We compared and contrasted ${\hat{C}}^{F}$ and *C* ^80^. If the numbers were similar, then the clusterPower simulations approximate the Murray equations and ${\hat{C}}^{\mathrm{cv}}$ could be used with confidence. If they were substantially different, further analysis would be required before evaluating ${\hat{C}}^{\mathrm{cv}}$.

${\hat{C}}^{\mathrm{cv}}$ was analyzed with respect to *C* ^80^. From this analysis, we observed the effects of cluster size variation on the required number of clusters.

## Results

### Without cluster size variability, simulations approximate formula

**Fig 4:**
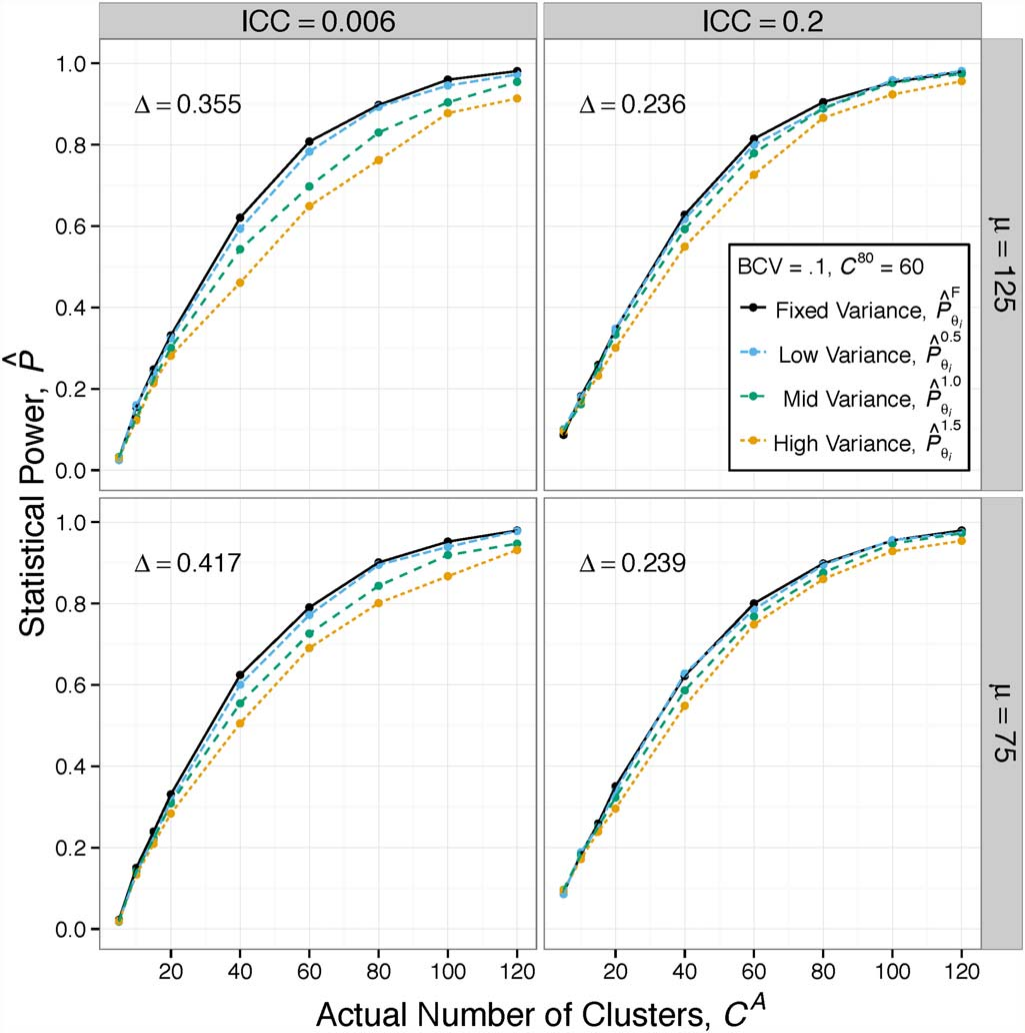
The CRT power curves for two different parameter sets over four levels of cluster size variance. When *C* ^*A*^ = 60, both fixed cluster size power estimates (${\hat{P}}_{\theta_{i}}^{F}$), the solid, black lines) should equal 0.8. By looking below the point (60, 0.8), one observes that power is lost as the cluster size variance increases in both scenarios. By looking to the right of the point, the observer notices that more clusters are required to attain a statistical power of 0.8 with increased variability.

The first clusterPower simulation used each unique parameter set (***θ***_***i***_) across a range of sample sizes (actual number of clusters, *C* ^*A*^) to generate fixed cluster size power estimates (${\hat{P}}^{F}={\hat{P}}_{\theta_{i}}^{F}(C^{A})$). These estimates were plotted to form the power curves represented by the black lines in Fig. 4. Using these results and the technique described in Methods Step 4, we calculated the number of clusters required to achieve a statistical power of 80% for fixed-size cluster sets (${\hat{C}}^{F}$). To validate clusterPower, ${\hat{C}}^{F}$ was compared to Murray’s formula-based estimate of clusters required to reach 80% power (*C* ^80^, from Equation 2).

The clusterPower simulations produced accurate, though conservative estimates. One example of this is the result from the lower left corner of Fig. 4. In this scenario, the first five ***θ***_***i***_ parameters (type I error(*α*) = 0.05, mean cluster size (*μ*) = 75, ICC = 0.006, BCV = 0.1, *C* ^80^ = 60) require an effect size (*Δ*) of 0.417 when the cluster sizes are fixed. Using these parameters and a *C* ^*A*^ = 60, we should find that the statistical power is near 80%. The average statistical power across 5,000 simulations, ${\hat{P}}^{F}$, was 79.04%. Since this is smaller than 80%, from this point and the one at *C* ^*A*^ = 80 we can interpolate to find that ${\hat{C}}^{F}=61.74$. This is a slightly conservative result, in that the statistical power was less than one percent lower than expected and the required number of clusters were two greater than expected.

More than half of our simulated ${\hat{C}}^{F}$ values were closer to Murray’s *C* ^80^ than in this example. In situations where *C* ^*A*^ = *C* ^80^, the ${\hat{P}}^{F}$ ranged from 75.04% to 81.44% with a median of 79.52%. The difference between ${\hat{C}}^{F}$ and *C* ^80^ ranged from -1.55 to 8.28 with a median difference of 0.51 (with negative values indicating ${\hat{C}}^{F}<C^{80}$). The ${\hat{C}}^{F}$ and *C* ^80^ were highly correlated (*R* = 0.9982). A paired two-sample t-test showed that ${\hat{C}}^{F}$ was significantly larger than *C* ^80^ and that the average difference is within one cluster (Estimate: 0.81, CI: (0.69, 0.92)).

While ${\hat{C}}^{F}$ did not perfectly reflect *C* ^80^, the high correlation and generally conservative estimates suggest that clusterPower can be used to simulate variable cluster size CRTs.

### With cluster size variability, power decreases

The subsequent clusterPower simulations used ***θ***_***i***_ with variable cluster size sets of *C* ^*A*^ clusters and three levels of coefficient of variance (*cv* = 0.5, 1.0, 1.5) to generate variable cluster size power estimates (${\hat{P}}^{cv}={\hat{P}}_{\theta_{i}}^{cv}(C^{A})$). These estimates were plotted to form the power curves that form the blue, green, and orange lines in Fig. 4 (which represent *cv* = 0.5, 1.0, 1.5, respectively). By performing the same process for calculating ${\hat{C}}^{F}$, we derived the number of clusters required to achieve a statistical power of 80% for variable cluster size sets, ${\hat{C}}^{\mathrm{cv}}$.

Using the lower left plot again as an example, one can see how power decreases and required number of clusters increases with greater cluster size variation. Looking below the ${\hat{P}}^{F}$ point at (60, 0.79), we see that the power decreases as variance increases: ${\hat{P}}^{0.5}=0.77$; ${\hat{P}}^{1.0}=0.73$; and ${\hat{P}}^{1.5}=0.69$. Looking to the right of the ${\hat{C}}^{F}$ point at (61.74, 0.8), we see that more clusters are required in the presence of variation: ${\hat{C}}^{0.5}=64.55$; ${\hat{C}}^{1.0}=72.6$; and ${\hat{C}}^{1.5}=79.82$.

This is merely one example of a consistent pattern of decreasing power as cluster size variability increased. Using paired t-tests, we observed that the difference between ${\hat{C}}^{0.5}$ and *C* ^80^ was significantly greater than zero (Estimate: 2.19, CI:(2.05, 2.34)); that ${\hat{C}}^{1.0}$ was significantly greater than ${\hat{C}}^{0.5}$ (Estimate: 4.09, CI:(3.82, 4.36)); and ${\hat{C}}^{1.5}$ was significantly greater than ${\hat{C}}^{1.0}$ (Estimate: 4.91, CI:(4.59, 5.23)).

Within each *cv* level, there is substantial variability in statistical power and required number of clusters due to the large diversity of parameter sets. We can observe this by looking at the percent increase in number of clusters required by variable cluster size sets over those from the Murray equation (${\hat{C}}^{\mathrm{cv}}\%$, from Equation 7). The range for ${\hat{C}}^{0.5}\%$ spans −2.03% to 30.83%, with a median of 7.47%; ${\hat{C}}^{1.0}\%$ covers 2.8% to 43.27%, with a median of 21.49%; and ${\hat{C}}^{1.5}\%$ ranges from 13.04% to 63.72%, with a median of 38.38%.

In 21 cases (5%), ${\hat{C}}^{0.5}$ was less than both the equivalent *C* ^80^ and *C* ^*F*^ values. However, there does not seem to be a common link between these scenarios, and thus their occurrence may be due to sampling variation from the stochastic simulations.

## Discussion

This study demonstrates that variability in cluster sizes reduces the power of a cluster-randomized trial when compared to a trial with no variation in cluster sizes. We observed between a 2% decrease and a 64% increase in the number of clusters needed across all scenarios studied. As the variability in cluster sizes increases, additional clusters are needed to maintain 80% power. This phenomenon has been described before [4, 6, 8, 10]. Our simulation study has confirmed these results, and allowed us to quantify the expected loss of efficiency across many different possible study design scenarios. These results may only hold for equal-armed cluster-randomized trials that have a continuous outcome measure and no controlled comparison.

A key feature of this paper is demonstrating the utility of the clusterPower package for R as a tool for conducting controlled simulation experiments to answer questions about CRT design. In this paper, we focused on estimating power in CRT designs with varying cluster sizes, but we have provided a template of a simulation study that could be used to answer many other types of questions. For example, different designs or analysis methods could be compared to determine the most efficient strategies for implementing CRTs.

Our simulations do not fix the overall sample size of a given study with variable cluster sizes. So the estimated powers within a given parameter set reflect, to some extent, different total sample sizes of the studies. However, these differences are averaged out across the many simulations. In general, this setting mimics situations where the cluster sizes cannot be controlled by investigators. This might be the case when, for example, health care workers at different-sized clinics will be enrolled in a study; or, students in different-sized classes will be enrolled in a study. In these situations, the investigator will not know the exact number of participants who will be enrolled in the study, just how many clinics and the average number per clinic.

Another difficulty that we did not confront in this paper is the effect of purposefully distributing different clusters into the treatment and control group. All group assignment was done in a random fashion. The impact of putting large clusters into one group and small clusters into the other is still unknown. Using some controlled comparison technique (such as matching on cluster size) may increase the efficiency of studies, but these methods were not examined in the present study.

Due to the experimental design, we cannot observe the impact that changing any of the CRT parameters may have upon the required number of clusters because all of them are used to calibrate the effect size.

As the use of cluster-randomized trials continue to expand in many scientific disciplines, it is vital that we continue to build our knowledge about how to design these trials efficiently. The results presented in this paper demonstrate the value of a new method for the efficient design of cluster-randomized trials in the presence of cluster size variability.
